# Actigraphic Assessment of Motor Activity in Acutely Admitted Inpatients with Bipolar Disorder

**DOI:** 10.1371/journal.pone.0089574

**Published:** 2014-02-20

**Authors:** Karoline Krane-Gartiser, Tone Elise Gjotterud Henriksen, Gunnar Morken, Arne Vaaler, Ole Bernt Fasmer

**Affiliations:** 1 Department of Neuroscience, the Norwegian University of Science and Technology, Trondheim, Norway and Department of Psychiatry, St. Olav’s University Hospital, Trondheim, Norway; 2 Department of Clinical Medicine, Section for Psychiatry, Faculty of Medicine and Dentistry, University of Bergen, Bergen, Norway, Division of Mental Health Care, Valen Hospital, Fonna Regional Health Authority, Norway and MoodNet Research Group, Division of Psychiatry, Haukeland University Hospital, Bergen, Norway; University of Iowa Hospitals & Clinics, United States of America

## Abstract

**Introduction:**

Mania is associated with increased activity, whereas psychomotor retardation is often found in bipolar depression. Actigraphy is a promising tool for monitoring phase shifts and changes following treatment in bipolar disorder. The aim of this study was to compare recordings of motor activity in mania, bipolar depression and healthy controls, using linear and nonlinear analytical methods.

**Materials and Methods:**

Recordings from 18 acutely hospitalized inpatients with mania were compared to 12 recordings from bipolar depression inpatients and 28 healthy controls. 24-hour actigraphy recordings and 64-minute periods of continuous motor activity in the morning and evening were analyzed. Mean activity and several measures of variability and complexity were calculated.

**Results:**

Patients with depression had a lower mean activity level compared to controls, but higher variability shown by increased standard deviation (SD) and root mean square successive difference (RMSSD) over 24 hours and in the active morning period. The patients with mania had lower first lag autocorrelation compared to controls, and Fourier analysis showed higher variance in the high frequency part of the spectrum corresponding to the period from 2–8 minutes. Both patient groups had a higher RMSSD/SD ratio compared to controls. In patients with mania we found an increased complexity of time series in the active morning period, compared to patients with depression. The findings in the patients with mania are similar to previous findings in patients with schizophrenia and healthy individuals treated with a glutamatergic antagonist.

**Conclusion:**

We have found distinctly different activity patterns in hospitalized patients with bipolar disorder in episodes of mania and depression, assessed by actigraphy and analyzed with linear and nonlinear mathematical methods, as well as clear differences between the patients and healthy comparison subjects.

## Introduction

Alterations in motor activity are important clinical features of acute bipolar disorder; mania is associated with overactivity, whereas depression usually is characterized by a reduction in activity [1]. Today, changes in psychomotor activity are assessed by the clinician, but objective recordings of activity could be useful for monitoring symptom severity and phase shifts in bipolar disorder.

In bipolar depression, psychomotor retardation and low mood are often more pronounced in the morning than in the evening [2], [3], implying a phase-delayed peak of activity [4]. Psychomotor agitation can occur in both depression and mania [5] and can be defined as motor restlessness with either goal-directed movements or sustained fidgeting and frequent position changes [6].

With the ongoing debate on the classification of bipolar disorders including bipolar spectrum disorders [7], psychomotor activation may become a key factor in separating subtypes. Actigraphy is a method of activity monitoring used in several studies in psychiatric populations [8]-[10]. An actigraph is a wrist-worn, relatively cheap and handy device, clinically useful in the evaluation of sleep disorders [11], [12]. Its role as an indicator of mood state is uncertain, but promising research findings [13]-[16] suggest that actigraphs might become a valuable supplement to clinical diagnostics.

However, little is known about actigraphically registered activity characteristics across different phases of bipolar disorder. The activity patterns in patients with major depression seem to be different from those of patients with schizophrenia and healthy controls [14], [17]. Other actigraphy studies have confirmed the clinical impression that patients with depression are less active during daytime, and that activity increases with treatment [10], but to our knowledge no studies have investigated differences in activity by actigraphy in acute episodes of bipolar disorder. Also, very few actigraphy studies have used adequate analytical methods to extract all relevant features from actigraphy data, irrelevant of study population [10]. In recent years it has been found that mathematical techniques with a theoretical basis in nonlinear dynamics may be used to describe the complexity seen in behavioral patterns [15], [18], [19].

For these reasons we aimed to compare patterns of motor activity during psychiatric hospitalization due to either mania or depression in bipolar disorder with healthy controls. Using 24-hour actigraphy recordings, we wanted to describe overall patterns of activity in these three groups and compare active periods in the morning and evening, by employing linear and non-linear mathematical methods [15].

## Materials and Methods

### Patients

Consecutively, acutely admitted inpatients at the Department of Psychiatry, St. Olav’s University Hospital, Trondheim, Norway, were asked to participate in a study assessing symptoms of agitation during admission. All Norwegian acute psychiatric services are public. All patients above 18 years in the catchment area who suffer from any acute psychiatric condition and are in need of acute admittance are admitted to this department. The only exclusion criterion was inability to give an informed consent in the primary examination by a senior psychiatrist or specialist in clinical psychology the first day after admittance. A total of 424 admissions were included in the study. If patients were admitted more than once, up to three admissions could be included. The patients with an inpatient stay of more than one day after inclusion were asked to wear an actigraph for 24 hours. In total, 280 actigraphy recordings were effectuated during hospitalizations between September 1^st^, 2011 and March 31^st^, 2012. The largest diagnostic group was affective disorders, which included 110 admissions (39.3% of the 280 admissions). Of these, 33 admissions were due to a primary ICD-10 diagnosis of bipolar disorder (F31.1–F31.5, current episode manic or depression). The sample excluded patients in mixed episodes (F31.6), patients in remission (F31.7) and patients with other bipolar affective disorders (F31.8 and F31.9, bipolar II disorder and unspecified bipolar disorder).

20 admissions were due to a current manic episode (F31.1, current episode manic without psychotic symptoms, and F31.2, current episode manic with psychotic symptoms), and 13 admissions were due to a current episode of depression (F31.3, current episode mild or moderate depression, F31.4, current episode severe depression without psychotic symptoms, and F31.5, current episode severe depression with psychotic symptoms). Two patients were admitted twice; one patient was first admitted in an episode of depression, then in an episode of mania, and one patient was admitted twice with mania. Second admissions were excluded from analysis, so that all patients are represented with only one admission. One recording was incomplete and therefore excluded. Finally, a total of 18 recordings from patients with bipolar disorder in a manic episode were compared to 12 recordings from patients with bipolar disorder in an episode of depression.

### Comparison Subjects

The comparison group (n = 28) primarily consists of employees at the Department of Psychiatry, Fonna Regional Health Authority in a western region of Norway, who were recruited through oral and written presentations approved by the Regional Committee for Medical and Health Research Ethics of Western Norway. None of the subjects were diagnosed with an affective disorder, nor were any of them prescribed psychopharmacological drugs, and they had no disposition to psychiatric disorders in general. They wore an actigraph for at least 24 hours during the period March 13^th^, 2012 - June 6^th^, 2013, and this 24-hour recording was included in the current study. 18 of them wore the actigraph in spring or summer months (April – August). 15 healthy controls wore the actigraph on a weekday, 11 healthy controls during the weekend, and 2 were retired. Comparison subjects are from now on referred to as healthy controls, although they were not age- nor gender-matched to the patients. No information about the weight of the healthy controls is available, and the samples could therefore not be matched by BMI.

### Recordings of Motor Activity

Motor activity was recorded using an actigraph (Actiwatch Spectrum, Philips Respironics Inc., Murrysville PA, USA), which contains a piezoelectric accelerometer programmed to record the integration of intensity, amount and duration of movement in all directions. A corresponding voltage is produced and stored as an activity count in the memory unit of the actigraph. Patients and healthy controls wore the actigraph around the wrist of their choice and were instructed not to take it off during the next 24 hours. Which wrist was not noted, but previous studies have found only small differences between the left and right wrist [20], [21]. Patient recordings contained an average of 1364.8±203.5 minutes for analysis, ranging from 435 to 1446 minutes. All starting times were in the daytime hours, between 09h14 and 19h51 (mean 12h41±2h35). Healthy control recordings contained 1440 minutes from midnight to midnight, as they were 100% compliant in wearing the actigraph for 24 hours.

Activity counts were recorded for one minute intervals during 24 hours. Data were analyzed for the total time of recording. From each patient and healthy control we also selected actigraphy data from 6 AM to midnight and separated this time period in morning and evening epochs. Morning epochs were defined to occur between 6 AM and 3 PM, and evening epochs between 3 PM and midnight. Because many of the recordings contained periods of inactivity, we searched each recording for periods of continuous motor activity in the morning and evening. The active morning period was searched from the start of the series, and the active evening period from the end of the series. From each participant we selected the first period of 64 minutes not containing more than two consecutive minutes of zero activity counts. If there was no such period, we searched for sequences with no more than 3 consecutive minutes with zero activity, and if that was not found, sequences with at most 4 consecutive minutes with zero activity. In this way we were able to obtain 64-minute sequences from almost all of the participants. 64 minutes were chosen due to the Fourier analysis, which requires sequence lengths to be potencies of 2 (32, 64, 128…).

One patient with mania did not have a valid recording in the morning, and consequently no active 64-minute period could be calculated. Another patient with mania lacked a 64-minute sequence with at most 4 consecutive minutes with zero activity in the morning. Both patients were omitted from morning series analyses, reducing the group with mania to 16 patients. One healthy control lacked a 64-minute sequence with at most 4 consecutive minutes with zero activity in the evening and was therefore excluded from evening series analyses.

### Mathematical Analyses

From the activity counts in the actigraph software program (Actiware, version 5.70.1) we calculated means for the whole recording period and the 64-minute periods of continuous motor activity. We further calculated the standard deviation (SD) for each time series, which is an intra-individual measure of fluctuations in activity, the root mean square successive difference (RMSSD), which describes the difference in successive counts from minute to minute, and the RMSSD/SD ratio. For the 64-minute time periods we additionally calculated autocorrelation (lag 1) and performed a Fourier analysis. We also used two measures of complexity: sample entropy and symbolic dynamic analysis. All techniques represent distinct means of characterizing a series of data in time. The software used for the estimation of sample entropy and for the Fourier analysis was obtained from the Physio Toolkit Research Resource for Complex Physiologic Signals [22], see http://www.physionet.org.

### Autocorrelation at Lag 1

An autocorrelation function is a mathematical tool to measure the degree of relationship between observations that are *k* lags apart. The autocorrelation at lag 1 is the correlation of a time series with itself lagged one step, in this case from minute to minute. As such, values closer to one indicate a stronger correlation. Autocorrelation analyses were performed using SPSS version 20.0.

### Sample Entropy

For the analysis of sample entropy the data were normalized by transforming the time series to have sample mean 0 and sample variance 1. Sample entropy is a nonlinear measure, which indicates the degree of regularity (complexity) of a time series, and is the negative natural logarithm of an estimate of the conditional probability that subseries of a certain length (m) that match point-wise, within a tolerance (r), also match at the next point. We chose the following values, m = 2 and r = 0.2. Sample entropy was used since it can be employed with comparatively short time series (>50) and is robust with regard to outliers [23].

### Symbolic Dynamics

The time series were transformed into series of symbols according to the method described by Guzzetti et al. [24] and Porta et al. [25]. For the analyzed active morning and evening sequences, the difference between the maximum and minimum value was divided into 6 equal portions (1–6) and each value of the series was assigned a number from 1–6, such that the transformed time series consisted of a string of numbers from 1–6. To avoid the problem with outliers the maximum value was set at no more than the mean +3 times the SD, and the minimum value was set at no less than the mean –3 times the SD. The series were then divided into overlapping sequences of three consecutive numbers. The series thus contained 62 such sequences, and the number of different sequences was counted, giving an indication of the complexity of the time series [26].

For the symbolic dynamic analyses we also used an alternative method to analyze the data, described by the same authors [24], [25]. Each sequence of three consecutive numbers was assigned one of four symbols, according to the following rule: 1) a pattern with no variation (e.g. 333), 2) a pattern with only one variation: two consecutive symbols are equal and the remaining symbol is different (e.g. 331), 3) a pattern with two like variations, such that the 3 symbols either ascend or descend (e.g. 641 or 235) and 4) a pattern with two unlike variations, both ascending and descending values (e.g. 312 or 451). The rates of occurrence of these four patterns were counted and the results given as the percentage of the total number of sequences analyzed (n = 62).

### Fourier Analysis

Data were normalized before analysis. No windows were applied. Results are presented as the relation between variance in the high frequency part of the spectrum (0.0021–0.0083 Hz, corresponding to the period from 2–8 minutes) and the low frequency part (0.00026 - 0.0021 Hz, corresponding to 8–64 minutes).

### Statistics

Statistical analyses were carried out using SPSS version 20.0. Means and standard deviations were calculated for the continuous variables, and proportions for the categorical variables. For comparison of means between patients we used t-tests, and for comparison of counts of categorical data chi-square tests. For comparison of subject characteristics and activity variables in all three groups, we used one-way ANOVAs with Bonferroni post-hoc tests to obtain differences between groups. A P-value ≤0.05 was considered significant.

### Ethics Statement

The patient study has been approved by the Regional Committee for Medical and Health Research Ethics of Central Norway, and the healthy control study by the Regional Committee for Medical and Health Research Ethics of Western Norway. All participants signed a written informed consent form prior to inclusion. The patients’ capacity to consent was established by a senior psychiatrist or specialist in clinical psychology, and patients who were unable to consent were not included in the study.

## Results

Subject characteristics are shown in table 1 and table 2. A significantly larger proportion of the group with mania than with depression was admitted to a psychiatric intensive care unit (PICU). Psychotropic drug treatment for both groups is summarized in table 2. Controls were more often employed and had a higher level of education than both patient groups (table 1). Controls and patients with depression were younger than the patients with mania (table 1).

**Table 1:** Demographic data, all groups.

Variable	Mania	Depression	Healthy controls
Number of subjects (n)	18	12	28
Age (years) (mean ± SD)	51.2±15.4	39.9±15.6	41.7±11.6*
Gender (female)	11 (61%)	7 (58%)	13 (46%)
Unemployment (incl. sick leave, retirement)	16 (89%)	9 (75%)	0###
Higher education (above high school)	6 (33%)	3 (25%)	19 (68%)###

If not mentioned otherwise, values are number of subjects, n (%).

*p≤0.05 one-way ANOVA. No significant differences between groups with Bonferroni post hoc test.

### p≤0.001 chi-square test where both patient groups were compared to healthy controls.

**Table 2:** Patient characteristics, groups according to current episode.

Variable	Mania	Depression
Number of patients (n)	18	12
Days admitted (mean ± SD)	22.9±17.2	22.2±24.6
Number of days between admission and actigraphy recording (mean ± SD)	2.1±1.1	3.5±2.7
Compulsory admission	9 (50%)	2 (17%)
Intensive care unit	10 (56%)	1 (8%)**
Body Mass Index (mean kg/m^2^± SD)	27.0±5.8	23.6±3.2
**ICD-10 primary diagnosis**	**n (%)**	**n (%)**
F31 Bipolar affective disorder		
- F 31.1, current episode manic without psychotic symptoms	7 (39)	–
- F 31.2, current episode manic with psychotic symptoms	11 (61)	–
- F 31.3, current episode mild or moderate depression	–	6 (50)
- F 31.4, current episode severe depression without psychotic symptoms	–	5 (42)
- F 31.5, current episode severe depression with psychotic symptoms	–	1 (8)
**Treatment**	**n (%)**	**n (%)**
- Antipsychotics	15 (83)	5 (42)
- Hypnotics/anxiolytics	9 (50)	6 (50)
- Anticonvulsants	7 (39)	4 (33)
- Lithium	2 (10)	1 (8)
- Antidepressants	1 (5)	1 (8)
- No psychotropic drug treatment	1 (5)	1 (8)

If not mentioned otherwise, values are number of patients, n (%).

**p≤0.01 chi-square test.

**Figure 1:**
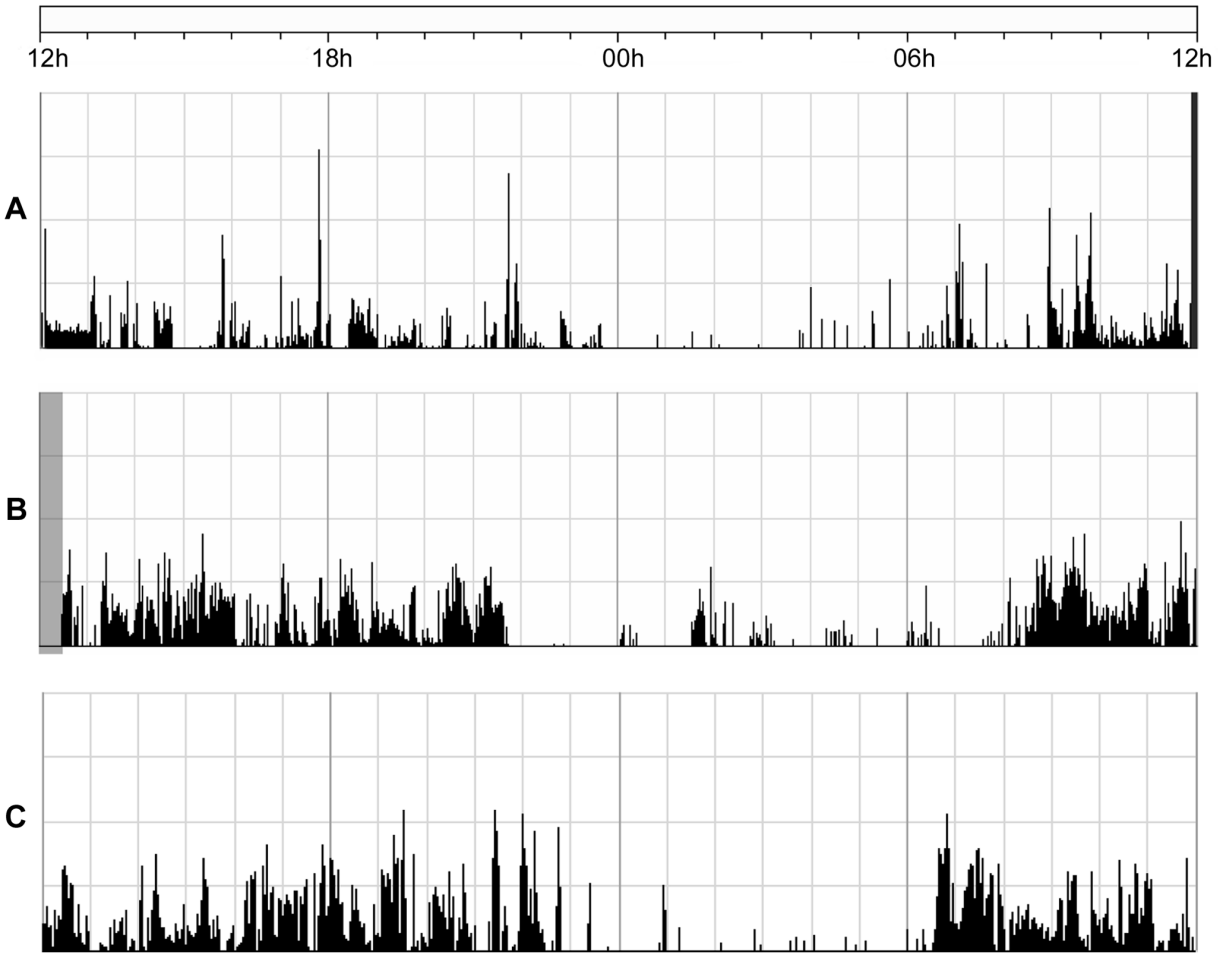
24-hour actigraphy recordings for all three groups. Activity counts from a patient with depression (A), a patient with mania (B) and a healthy control (C). The figure shows the activity counts in black during 24 hours from 12 h to 12 h the next day. The dark gray area at the end of recording A and the gray area at the beginning of recording B represent excluded periods, when the subject was not wearing the actigraph.

24-hour actigraphy recordings for a patient with depression, a patient with mania and a healthy control subject are shown in Figure 1. Analysis of the 24-hour recording shows a lower mean activity level for the patients with depression compared to the healthy controls (table 3), and the RMSSD in percent of mean activity is higher for the depression group compared to the controls. Both patient groups also have a significantly higher RMSSD/SD ratio compared to healthy controls.

**Table 3:** Results from the 24-hour recording of motor activity.

Variable	Mania	Depression	Healthy controls	p-value
Number of subjects (n)	18	12	28	
Mean/minute	157±84	128±76+	203±71	**0.014**
SD/min in % of mean	145.1±39.1	179.4±56.9	147.3±29.8	**0.043**
RMSSD/min in % of mean	113.7±41.8	150.5±62.4+ (T)	99.1±21.5	**0.002**
RMSSD/SD	0.774±0.094***	0.828±0.098+++	0.675±0.077	**<0.001**

All data are given as mean ± SD. p-values obtained in a one-way ANOVA. Post hoc Bonferroni tests:

***p≤0.001, mania compared to healthy controls.

+ p≤0.05, depression compared to healthy controls.

+++ p≤0.001, depression compared to healthy controls.

(T): Tamhane’s T2 post hoc test was used when unequal variances were assumed.

**Figure 2:**
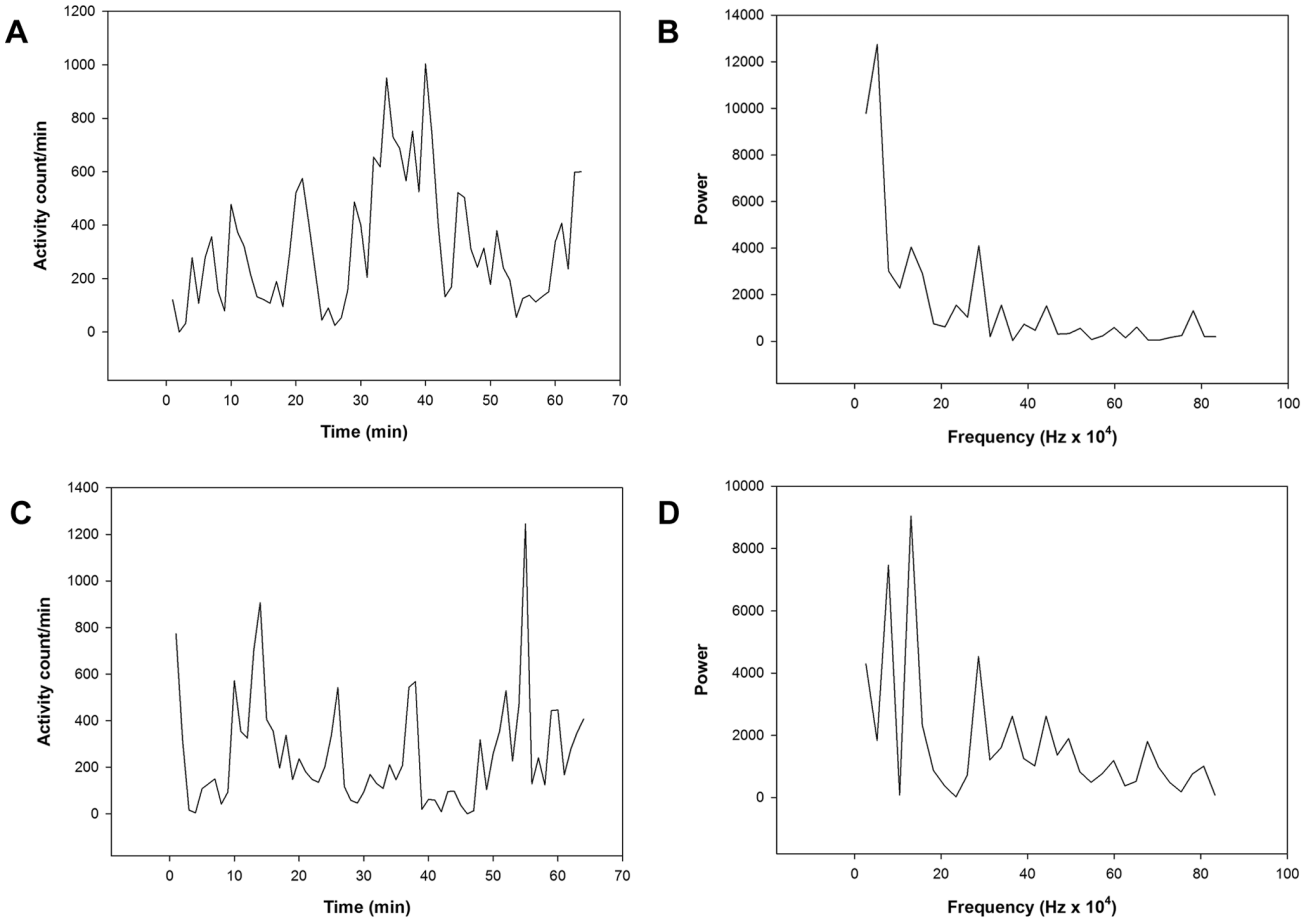
Actigraphy recordings of 64-minute periods of continuous motor activity in the morning. Activity counts and Fourier analysis from a healthy control (A and B) and a patient with mania (C and D).

Activity variables for the 64-minute period of continuous motor activity in the morning, also referred to as the active morning period (Figure 2), are shown in table 4. The motor activity is significantly lower in patients with mania and depression compared to healthy controls. The SD in percent of mean activity is significantly higher in the depression group compared to both patients with mania and healthy controls. The RMSSD in percent of mean activity is also higher in the patients with depression compared to both other groups, but only significantly different from the healthy controls. Patients with mania have the highest RMSSD/SD ratio, also significantly different from the healthy controls. Compared to the healthy controls, patients with mania have a significantly lower autocorrelation in the first lag, and a significantly higher ratio between variance in the high-frequency and the low-frequency parts of the spectrum, as shown by the Fourier analysis (Figure 2). The symbolic dynamic analysis shows the highest number of unique sequences in the mania group, and the difference between the two patient groups is significant. The alternative method of symbolic dynamic analysis also shows the highest complexity in patients with mania, but the differences between the groups are not significant (data not shown). Similarly, the patients with mania have the highest value for sample entropy (table 4).

**Table 4:** Results from the 64-minute period of continuous motor activity in the morning.

Variable	Mania	Depression	Healthy controls	p-value
Number of subjects (n)	16	12	28	
Mean/minute	215±144***	235±101++	391±139	**<0.001**
SD/min in % of mean	87.3±21.7	117.0±41.9^+#^	89.4±24.3	**0.012**
RMSSD/min in % of mean	86.1±28.6	107.4±46.3++	74.7±23.1	**0.012**
RMSSD/SD	0.980±0.178*	0.919±0.172	0.844±0.140	**0.026**
Sample entropy (m = 2, r = 0.2)	1.474±0.624	1.032±0.481	1.114±0.407	**0.034**
Symbolic dynamics	36.81±6.25	30.08±6.54#	33.61±6.57	**0.031**
Fourier analysis	0.87±0.48*	0.70±0.34	0.55±0.27	**0.025**
Autocorrelation Lag 1	0.493±0.163**	0.561±0.163	0.628±0.116	**0.013**

All data are given as mean ± SD. p-values obtained in a one-way ANOVA. Post hoc Bonferroni tests:

*p≤0.05, mania compared to healthy controls.

**p≤0.01, mania compared to healthy controls.

***p≤0.001, mania compared to healthy controls.

+ p≤0.05, depression compared to healthy controls.

++ p≤0.01, depression compared to healthy controls.

# p≤0.05, depression compared to mania.

Regarding the active evening period (table 5), the only significant difference is a higher RMSSD/SD ratio in the patients with mania compared to healthy controls.

**Table 5:** Results from 64-minute period of continuous motor activity in the evening.

Variable	Mania	Depression	Healthy controls	p-value
Number of subjects (n)	18	12	27	
Mean/minute	213±122	177±74	247±137	0.245
SD/min in % of mean	97.4±37.8	123.8±44.0	112.5±41.7	0.216
RMSSD/min in % of mean	99.1±46.9	117.1±47.4	96.4±39.0	0.379
RMSSD/SD	1.010±0.171*	0.949±0.142	0.866±0.166	**0.017**
Sample entropy (m = 2, r = 0.2)	1.309±0.703	1.083±0.440	0.976±0.516	0.165
Symbolic dynamics	33.72±7.51	28.92±7.53	31.30±8.65	0.281
Fourier analysis	1.08±0.71	0.77±0.34	0.72±0.59	0.126
Autocorrelation Lag 1	0.470±0.171	0.538±0.128	0.538±0.167	0.080

All data are given as mean ± SD. p-values obtained in a one-way ANOVA. Post hoc Bonferroni test:

*p≤0.05, mania compared to healthy controls.

When the patients with mania are separated into two groups; one including patients admitted to a PICU (n = 10) and one including patients admitted to an ordinary ward (n = 8), the patients in the PICU have a significantly increased RMSSD/SD ratio, lower autocorrelation in the first lag and higher variance in the high frequency part of the spectrum in the Fourier analysis, in the active morning period only. Also, in the 24-hour recording they have an increased SD in percent of mean activity compared to patients on an open ward (data not shown).

## Discussion

The main findings of our study are distinct differences in motor activity parameters between acutely hospitalized patients with bipolar disorder in an episode of mania or depression. Compared to healthy controls there are also clear differences. In addition we find a distinct diurnal pattern, with the most pronounced differences during periods with continuous activity in the morning.

The patients with depression are less active than healthy controls over 24 hours, which corresponds with low activity being a common factor in depression [10]. Also, both patient groups are less active than the healthy controls during a 64-minute period of continuous activity in the morning, which probably partly reflects a natural consequence of staying on a hospital ward within a limited area, and partly the effect of psychotropic drug treatment. Without these constraints the patients with mania would be expected to be more active than the healthy controls [27], [28].

Thus, while the total activity counts do not distinguish between patients in a manic and a depressive phase, other parameters show differences in regulation of motor activity in bipolar mania and depression. For the patients with depression the reduced activity level seems to be characterized by increased variability, as shown by the significantly increased standard deviation (SD) in an active period in the morning compared to both patients with mania and healthy controls. In addition, the patients with depression have a significantly higher root mean square successive difference (RMSSD) over 24 hours and in the active morning period compared to healthy controls. These findings are in agreement with a previous study on motor activity of patients with depression [15], and increased intra-individual variability has also been found in actigraphy studies of ADHD patients [29].

Interesting is also the increased RMSSD/SD ratio in the patient groups compared to the healthy controls, meaning that the alteration between successive activity counts increases relative to overall variability. For the patients with depression this ratio is only significantly different from healthy controls in the 24-hour recording, whereas the patients with mania have a higher ratio compared to healthy controls in all analyzed time series. An increased RMSSD/SD ratio has been found in patients with schizophrenia [15] and in healthy individuals treated with the glutamatergic N-methyl-D-aspartate (NMDA) antagonist memantine [30].

Autocorrelation in the first lag was significantly lower in the patients with mania compared to controls in the active morning period, which indicates less correlation of activity counts from minute to minute and corresponds well with the increased RMSSD/SD ratio. A lower autocorrelation between adjacent activity recordings has also been associated with memantine and was in addition found in a sample of patients with schizophrenia [30].

The Fourier analysis shows that the patients with mania demonstrate an increased variance in the high frequency part of the spectrum in the active morning series, corresponding to the period from 2–8 minutes. This was also found in a sample of patients with schizophrenia [15], and although it was considered to reflect a fundamental difference between schizophrenia vs. depression and no mental illness, our results indicate that it could be a common factor of both schizophrenia and mania. In the memantine-treated individuals a higher variance in the high frequency compartment of the spectrum was found as well [30].

The pattern that thus emerges from these different analyses related to variability is that bipolar depression is characterized by generally increased variability, reflected in the SD and RMSSD analyses, while mania is characterized by increased variability in the high frequency part of the spectrum, corresponding to the 2–8 minute period in the Fourier analysis, and is also seen in the increased RMSSD/SD ratio and the reduced autocorrelation (lag 1).

The group with mania also show increased complexity compared to patients with depression in the symbolic dynamic analyses of the active morning periods. The results from the sample entropy analyses closely correspond to this finding, supporting the notion that a manic state is accompanied by increased complexity of motor activity patterns. An increase in entropy indicates a higher level of disorder and unpredictability in a time series. Again, a similar increase in sample entropy has been found in patients with schizophrenia [15] and was suggested to represent a partial breakdown in structured normal activities of everyday life. Reduced complexity in different physiological systems has been postulated to be characteristic of both diseases and aging processes [31]. However, this may depend on the dynamics of the system under study. Vaillancourt and Newell [32] have postulated that in systems with intrinsic oscillations, disease processes may instead be accompanied by increased complexity. In addition to the findings from patients with schizophrenia referred to above, more random stride-interval fluctuations in aging and in patients with Huntington’s disease has been reported by Hausdorff et al [33], and increased complexity has been described in the EEG patterns of patients with mania [34].

Our findings for the group with mania closely correspond to the findings in patients with schizophrenia in a study from Bergen, Norway, where the same mathematical analytical methods were employed to analyze actigraphy data of patients with schizophrenia and depression [15]. Also, an associated group of investigators found that memantine induced movement patterns which partly resembled those found in patients with schizophrenia and suggested that the NMDA receptor could be involved in movement disturbances in schizophrenia [30]. Bipolar disorder has also been associated with a dysfunctional frontal glutamate system [35]. Based on the present study the question of how the NMDA receptor may be involved in mania-associated activity patterns should be further explored.

Although the patients with depression demonstrate reduced complexity compared to the patients with mania, using symbolic dynamic analyses, the values are not significantly lower when compared to healthy controls. It would, however, be interesting to study this further in other groups of patients with depression, since Friedman et al [36] found reduced complexity of mesolimbic dopaminergic activity in a rat model of depression.

The highly significant differences between the three groups despite only 12 and 18 patients in the patient groups and 28 healthy controls indicate clear differences in the regulation of motor activity between the three groups.

There are some limitations to our study which may restrict the findings: The number of participants is rather small, and psychotropic drug treatment may have biased the results; a greater proportion of the patients in an episode of mania were prescribed antipsychotic medication. On the other hand, the marked diurnal variation observed, with significant differences between the groups mainly in the active morning sequences argue against drug treatment as a main reason for the present results.

The fact that more patients with mania were admitted to a psychiatric intensive care unit (PICU), may have affected the results. This could be an alternative explanation of the similarities between our patients in an episode of mania and the patients with schizophrenia described previously, and is supported by the finding that the patients in a PICU, compared to the patients on an ordinary ward, have values of RMSSD/SD, autocorrelation and Fourier analysis which are similar to results from patients with schizophrenia. A PICU implies a single room under regular surveillance, and all patients are allowed to move about freely, but within a more limited space in the intensive care unit than on an open ward.

It is possible that hospitalization in itself can explain the differences in activity between patients, as hospital programs and schedules may have increased activity for the patients with depression and restricted the patients with mania. However, because the total 24-hour activity counts do not separate the patients with mania and depression, the differences found in other measures seem all the more significant, as they cannot be attributed to differences in total activity levels.

Activity is expected to differ between workdays and weekends for non-hospitalized individuals. Dividing the healthy controls in 15 who wore the actigraph on a weekday and 13 who were monitored in the weekend or were retired, produces only one significant difference in all analyses: RMSSD/SD for the 24-hour period. We therefore assume that the day when the actigraph was worn cannot account for the differences in activity between the controls and the patients.

The seasons when the groups were monitored may also have biased the results, since 18 of the 28 healthy controls were monitored in spring or summer months, as opposed to the patients who were all monitored in fall or winter months. The healthy controls have completed the Seasonal Pattern Assessment Questionnaire (SPAQ) [37], and the majority of controls report a moderate seasonal change in energy level, with the most energy during spring or summer. On the other hand, studies suggest that activity may not show seasonal variation, possibly because activity seems to be less influenced by light exposure than mood [38], [39].

Patients were all hospitalized in an acute state of their bipolar disorder, and results should therefore not be generalized to this diagnostic group as a whole. It is possible that hospitalized patients will have different activity profiles than outpatients with a less severe course of illness.

In tables 3 to 5, several ANOVA tests were conducted on activity measures from the data. A correction for multiple comparisons adjusting for the total number of statistical tests has not been done since the analyses were planned before they were conducted [15], [30], [40].

Despite the limitations mentioned, the main findings of our study remain well-founded: episodes of bipolar depression are associated with reduced 24-hour activity and an activity pattern characterized by high intra-individual variability. Acute bipolar mania is also associated with lower mean activity levels than non-hospitalized healthy individuals, but less variance in activity compared to patients with depression. In addition, a more complex activity pattern is found in patients with mania, and at the same time Fourier analysis shows increased variability in the high frequency part of the spectrum, and probably related to this, an increased RMSSD/SD ratio and reduced autocorrelation (lag 1).

Our results provide strong indications that psychomotor function shows opposite symptom manifestations in acutely admitted patients with bipolar depression and mania, and that these manifestations can be identified by actigraphy recordings analyzed with linear and nonlinear methods. Further research on larger samples and other psychiatric populations is warranted, as it could establish the role of actigrahy as a diagnostic and therapeutic marker of psychiatric disorders in clinical practice.
